# Ocular melanoma in a patient successfully treated for diffuse malignant peritoneal mesothelioma: a case report

**DOI:** 10.1186/1477-7819-10-90

**Published:** 2012-05-21

**Authors:** Sara Langlais, Juan Pablo Velazquez-Martin, Pierre Dubé, Ernest Rand Simpson, Guy Leblanc, Lucas Sideris

**Affiliations:** 1 Department of Surgery, Hôpital Maisonneuve-Rosemont, Université de Montréal, 5415 Boulevard de l’Assomption, Montréal, H1T 2 M4, QC, Canada; 2 University of Toronto, Princess Margaret Hospital/University Health Network, 610 University Avenue, Toronto, M5G 2 M9, ON, Canada

**Keywords:** BAP1, Diffuse malignant peritoneal mesothelioma, Ocular melanoma

## Abstract

****Background**:**

Diffuse malignant peritoneal mesothelioma and ocular melanoma are both rare tumors. To the best of our knowledge there is only one previous report of three cases in a family with known susceptibility to malignancies associating diffuse malignant peritoneal mesothelioma and ocular melanoma, with no sporadic cases previously reported.

****Case presentation**:**

We describe the case of a 59-year-old man with a history of diffuse malignant peritoneal mesothelioma, who presented with ocular melanoma 41 months after cytoreductive surgery and hyperthermic intraperitoneal chemotherapy. We also briefly review the literature.

****Conclusions**:**

Diffuse malignant peritoneal mesothelioma is an uncommon but aggressive disease. As diffuse malignant peritoneal mesothelioma characteristically remains confined to the abdominal cavity, any new extra-abdominal symptom should eventually raise suspicion of another primary tumor. Few cases of diffuse malignant peritoneal mesothelioma associated with other primary tumors have been reported. As ocular melanoma is also infrequent, we suspect a genetic predisposition to these tumors. There is emerging evidence supporting the role of *BAP1* mutations in the pathogenesis of these two neoplasias.

## **Background**

Diffuse malignant peritoneal mesothelioma (DMPM) is a rare but aggressive tumor arising from the serosal lining of the peritoneal cavity. In industrialized countries, incidence rates of DMPM range between 0.5 and 1.7 cases per million in the general population [1]. Using traditional treatment modalities such as systemic chemotherapy and palliative surgery, median survival is poor (about 12 months). For nearly a decade, a new approach combining aggressive cytoreductive surgery (CRS) and hyperthermic intraperitoneal chemotherapy (HIPEC) has dramatically improved median survival, and this has reached 5 years in some series [2].

We present the case of a patient with DMPM initially treated with this approach, who later developed ocular melanoma (OM). Few other primary tumors have been linked to DMPM. To the best of our knowledge there is only one previous study reporting three patients with DMPM and OM, all in the same family [3].

## **Case presentation**

**Figure 1:**
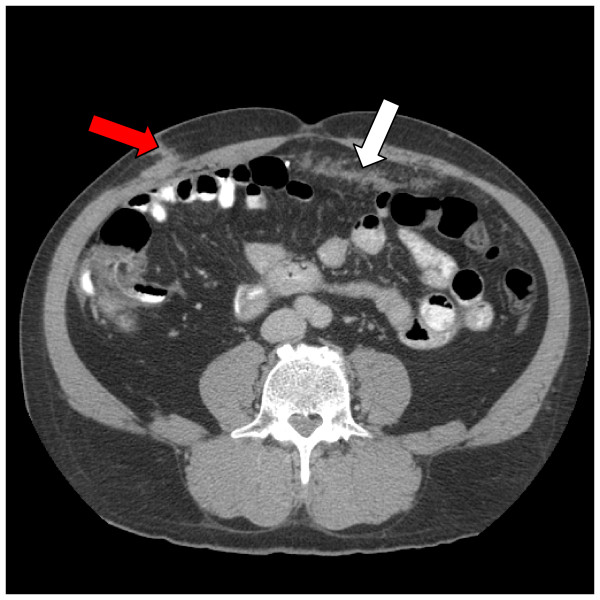
**Computed tomography (CT) scan of the abdomen.** This CT scan of the abdomen performed before cytoreductive surgery (CRS) and hyperthermic intraperitoneal chemotherapy (HIPEC) shows neoplastic involvement of trocar sites (red arrow) and omentum (white arrow).

A 59-year-old man sought medical attention in 2005 for vague abdominal discomfort, nausea and belching that had started 6 months earlier. The patient did not have any previous history of cancer in his family or any history of asbestos exposure. He denied any other gastrointestinal or constitutional symptoms. A physical examination was unremarkable except for a slightly distended abdomen. Initial investigations included routine laboratory investigations, an upper gastrointestinal tract endoscopy and a colonoscopy, all within normal limits. However, an abdominal computed tomography (CT) scan revealed omental caking, perihepatic ascites and neoplastic seeding in Morrison’s pouch and the perihepatic space (Figure 1). There was no evident lymphadenopathy present. A single hepatic lesion was further investigated by magnetic resonance imaging (MRI), which was compatible with a benign hemangioma. A staging investigation was negative for extra-abdominal metastases. The patient underwent diagnostic laparoscopy. Intraoperative findings revealed an omental thickening with nodules on the omentum and diffuse involvement of the peritoneal surface with relative sparing of the liver, spleen, stomach, and small and large intestine. Peritoneal biopsies results were compatible with DMPM of the epithelial subtype. He was then referred to our institution for definitive treatment. At 3 months after diagnostic laparoscopy, our team performed a complete CRS and HIPEC. Total right diaphragmatic, partial left diaphragmatic (2/3), total pelvic and parietal anterior peritonectomies were performed. The CRS procedure also included cholecystectomy, splenectomy, omentectomy, mesenteric implant fulguration and removal of laparoscopic surgery trocar sites that were invaded with tumor nodules. The peritoneal cancer index (PCI) was 21 (maximal possible score: 39). This index takes into account the number of invaded areas from a total of 13, and the maximal size of tumors nodules within 3 possible groups (<5 mm, 5 mm to 5 cm, >5 cm) [4]. After CRS, there was no residual tumor (completeness of cytoreduction (CCR) score = 0). HIPEC was performed with oxaliplatin 460 mg/m^2^ for 30 minutes and systemic 5-FU was simultaneously infused. The findings on the final pathological report were as follows: multifocal and multinodular tumor with numerous psammomatous calcifications, tumor made with polygonal cells showing a tubulopapillary proliferation pattern, cells present on the peritoneal surface and invading the underlying tissue inducing desmoplasia, tumor cells of intermediate size with a moderately abundant eosinophilic cytoplasm, round and slightly irregular nuclei with prominent nucleoli, weak mitotic activity, no intracytoplasmic mucin on periodic acid-Schiff diastase (PAS-D) staining. Immunohistochemical studies have been performed for vimentin, keratin 5/6, calretinin, Wilms' tumor 1 (WT1), carcinoembryonic antigen (CEA), CD15, E cadherin, Ber-EP4 and CD141. Tumor cells were extremely positive for calretinin, keratin 5/6 and focally positive for CD141. In conclusion, the histological aspect and the immunohistochemistry confirmed the diagnosis of DMPM of epithelial subtype.

Our patient’s postoperative course was complicated by respiratory failure episodes (secondary to pleural effusions and pulmonary edema), a right subphrenic abscess and a lower limb deep venous thrombosis. Recovery was slow but complete and our patient was discharged after 6 weeks. He was then followed with a positron emission tomography-computed tomography (PET-CT) scan every 4 months for 1 year and then every 6 months. At 36 months after treatment (June 2009), a PET-CT scan showed no evidence of recurrence.

**Figure 2:**
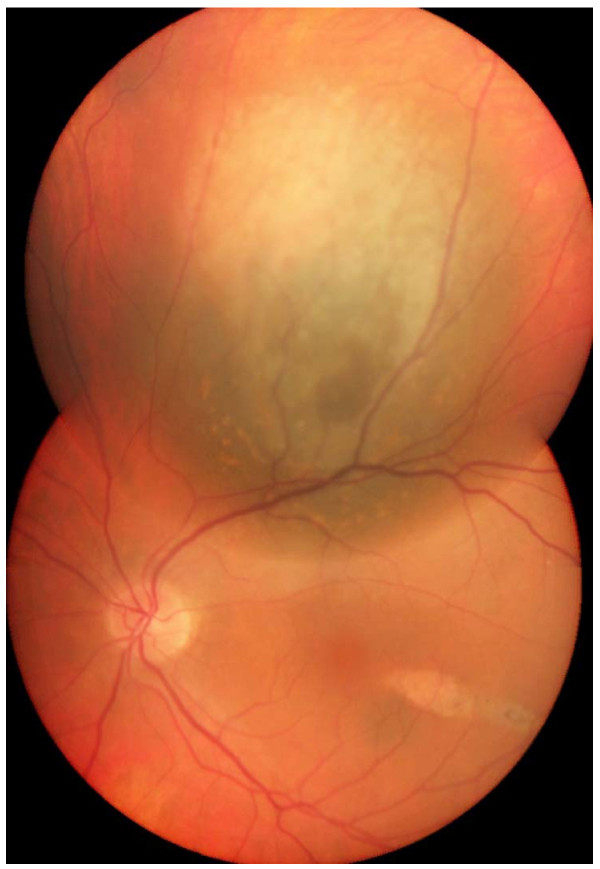
**Ocular fundus image of the choroidal melanoma.** Clinical appearance of the choroidal mass after receiving external radiation therapy. A choroidal melanocytic lesion was found superior to the macular area. Note the orange pigment, which is highly suggestive of choroidal melanoma.

**Figure 3:**
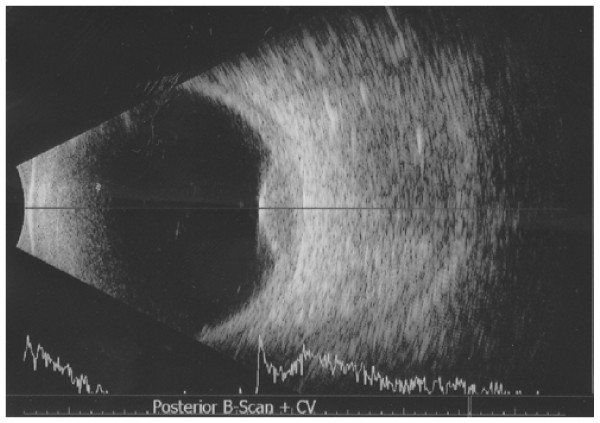
**Ultrasound of the left eye.** Combined A-scan and B-scan ultrasound showing an acoustically hollow dome-shaped lesion, which demonstrated a medium to low reflectivity, and a maximum height of 3.15 mm.

In November 2009 (41 months after CRS and HIPEC), the patient complained of blurred vision in his left eye and was referred to an ophthalmologist. Fundoscopic examination of the left eye revealed a solid pigmented choroidal mass above the macula measuring 6.9 × 9.2 mm in base and 2.5 mm in height. The patient was then referred to a medical oncology service for systemic staging. MRI of the brain, and a CT scan of the thorax, abdomen and pelvis did not show any evidence of metastatic disease. The medical oncology team assumed that the left eye tumor was metastatic disease from DMPM and external beam radiotherapy of a total dose of 40 Gy (20 fractions of 2 Gy) was administered. Further ophthalmologic assessment showed no treatment response, the size of the tumor increased to 8.1 × 11.3 mm in base and 2.9 mm in height. Two fine needle biopsies were performed but the results were deemed inconclusive. A second ophthalmologic evaluation by a specialized group of ocular oncologists was obtained. On fundus examination, a choroidal melanocytic lesion superior to the macular area was found. The lesion showed different grades of pigmentation, orange pigment on its surface and the presence of subretinal fluid (Figure 2). B-scan ultrasound revealed an acoustically hollow dome-shaped lesion (Figure 3), which on A-scan demonstrated a medium to low reflectivity, and a maximum height of 3.15 mm. Based on the clinical appearance and ancillary testing, a diagnosis of ocular melanoma was supported.

In November 2010, brachytherapy with an iodine-125 plaque at a total dose of 80 Gy to the apex of the tumor was performed. In October 2011 the tumor reduced in size to 2.9 mm in height and there was no sign of recurrence of DMPM or OM on PET-CT.

## **Discussion**

DMPM accounts for 30% of all malignant mesotheliomas [5]. The median age at diagnosis is 65 to 69 years. Asbestos exposure is the major risk factor for peritoneal mesothelioma even though association is less strong than for pleural mesothelioma, especially in women. Infection by the simian tumor virus (SV-40) and prior radiation exposure may also play a role in pathogenesis of DMPM [6].

DMPM can be divided into three histologic subtypes: epithelial (most common), sarcomatoid and biphasic (epithelial and sarcomatoid components). Immunohistochemistry is helpful to distinguish DMPM from other peritoneal cancers (colorectal adenocarcinoma and ovarian carcinoma, among others) [5]. Metastatic papillary adenocarcinoma was the initial diagnosis in this case, which was later confirmed to be a DMPM by immunohistochemical studies.

Although there is no commonly accepted standard of care for DMPM, it is well established that CRS and HIPEC therapy has prolonged median survival: 12 months with palliative surgery and/or systemic chemotherapy versus nearly 5 years with CRS and HIPEC [2].

In the investigation of new symptoms in DMPM cases, clinicians must recall that DMPM characteristically tend to recur locally and therefore, extra-abdominal metastases are uncommon. The hypothesis of another primary tumor should be considered despite the few cases reported of DMPM associated with primary malignancies, especially in the context of lacking a history of asbestos exposure as in our case. A series of 500 cases of DMPM reported a 1.8% rate of DMPM associated with other primary tumors [7]. In a multicenter study of 81 cases, a higher incidence (8.6%) was reported [8]. Various types of cancer have been previously associated with DMPM (colorectal, breast, multiple myeloma, bronchogenic and bladder carcinomas) but none with a strong association [7-9].

Similar to DMPM, OM is an infrequent malignancy with an estimated annual incidence of six cases per million. It is the most common primary intraocular malignancy in adults, with the choroid affected in 90% of cases [10]. Known risk factors include fair skin, light-colored eyes and the presence of pre-existing uveal nevus (our patient has the two former features) [11]. Several genetic mutations in chromosomes 3, 6 and 8 have also been identified in association with OM [11]. Current treatment includes brachytherapy for small and medium-sized tumors, with enucleation reserved for large tumors [12]. An overall survival of 50% at 10 years is accepted.

The specific association between OM and mesothelioma has been previously described in three cases in a family with known susceptibility to malignancies. All cases were found to have a *BAP1* mutation [3]. *BAP1* is a tumor suppressor gene located on chromosome 3p21 and the BAP1 protein is involved in DNA damage response, regulation of cell cycle and cell growth. Mutations in *BAP1* are infrequent in the general population but high in mesothelioma (20.1%) and uveal melanoma (44.1%) [13-15]. The joint probability of the occurrence of both mesothelioma and OM in the same individual by chance has been estimated as 36 per trillion per year by Testa *et al*. [3] In that study, the authors found germline *BAP1* mutations in 2 of 26 patients diagnosed with mesothelioma and both patients were previously diagnosed with uveal melanoma. Three of their four patients with uveal melanoma and *BAP1* mutations subsequently developed mesothelioma. They have suggested a new *BAP1*-related cancer syndrome characterized by mesothelioma and ocular melanoma, and possibly other malignancies [3]. In our patient, the mesothelioma was the presenting malignancy and 41 months later a choroidal melanoma was diagnosed. Clinicians should be aware of this entity since individuals who carry germline *BAP1* mutations may be at increased risk of developing metachronously both OM and DMPM. Patients found with *BAP1* mutations and their families should be closely monitored for the development of any or both of these malignancies for the possibility of early detection and intervention.

## **Conclusions**

Patients with DMPM who undergo CRS and HIPEC can expect a longer life expectancy in comparison to patients treated by other therapeutic modalities. Nevertheless, the association of other primary cancer with DMPM may complicate the management and alter the prognosis for these patients.

To the best of our knowledge, this is the first known case report of DMPM and OM occurring in the same individual with no family history. This article is an important element to support the concept of a clinical correlation between DMPM and OM, probably as a *BAP1*-related cancer syndrome since the probability of the occurrence of both malignancies by chance in the same individual is extremely remote. Genetic testing for *BAP1* mutations in this patient and his family is currently in progress and the results will be the subject of another publication.

## **Consent**

Written informed consent was obtained from the patient for publication of this case report and any accompanying images. A copy of the written consent is available for review by the Editor-in-Chief of this journal.

## Competing interests

The authors declare that they have no competing interests.

## Authors’ contributions

Design and writing: LS, SL. Critical review: VMJP, SER, DP, LG. Data collection: LS, VMJP, SL, DP. All authors read and approved the final manuscript.
